# Mixed-ownership reform and strategic choice of Chinese state-owned enterprises

**DOI:** 10.1371/journal.pone.0284722

**Published:** 2023-04-21

**Authors:** Runsen Yuan, Chunling Li, Xiaoran Sun, Nosherwan Khaliq

**Affiliations:** 1 School of Economics and Management, Yanshan University, Qinhuangdao, Heibei Province, PR China; 2 School of Management, University of Bristol, Bristol, United Kingdom; Alexandru Ioan Cuza University: Universitatea Alexandru Ioan Cuza, ROMANIA

## Abstract

The strategic choice of state-owned enterprises (SOEs) is crucial to the sustainable development of China’s economy. This paper explores the impact of mixed-ownership reform on the strategic choice of SOEs from the shareholder power and the board power. We find that the greater the diversity of mixed shareholders, the depth of mixed equity, the control of mixed equity, and the excess control of mixed equity, the higher the degree of mixed-ownership reform, and the more likely it is to promote SOEs to choose the prospector strategy. The mechanism test states that the impact of mixed-ownership reform on enterprise strategy is achieved through the balance effect between non-state-owned shareholders and state-owned controlling shareholders with the same power, and the synergy effect between different powers of non-state shareholders. Further research indicates that the mixed-ownership reform has a stronger driving effect on the prospector strategy in SOEs under strict external supervision, competitive industries, and local areas. This study clarifies the governance logic of non-state-owned shareholders on the strategic positioning of SOEs by dual control rights, and it provides empirical evidence for the formulation of enterprises’ market-oriented strategic objectives.

## 1. Introduction

As one of the core elements of strategic management theory, the strategic choice mainly provides customers with convenience or superior products and services through a series of activities to form a competitive advantage. With the sudden onset of COVID-19 in 2019, enterprises are faced with a highly volatile market competition environment. Many enterprises have already begun to adjust their strategic objectives to adapt to changes in the external environment. At the corporate strategy meeting in September 2021, Meituan proposed to upgrade its strategy from the "Food + Platform" to "Retail + Technology": (https://view.inews.qq.com/a/20220226A02JFS00?startextras=0_548c064703008&from=ampzkqw). The retail business has become the strategic emphasis in the next stage. This adjustment provides convenience for consumers’ lives under the regular epidemic prevention and control situation. It can be seen that strategic choice is closely related to the success or failure of enterprise operations. Although the epidemic will hit some industries, it may also promote the development of new businesses in some enterprises and give birth to new products and new markets. The crux to the different results lies in whether the governance subject of the enterprise can identify the strategic positioning timely in line with the changes in the environment and fully grasp the market opportunities. Miles et al. [1] illustrated that the difference in enterprise strategy choice is not due to their industries. On the contrary, enterprises will choose various strategies in any industry. The strategic choice of enterprises vary with the power structure and values of the governance subjects [2]. Then, when the external environment changes, how to optimize the enterprise’s governance structure and urging the governance subject to select an appropriate strategy is very significant for enterprises to achieve sustainable development in the fierce market competition.

The strategic choice that determines the enterprise’s overall long-term planning is affected by many factors. It mainly includes external environmental factors such as political environment [3], market mechanism [4], industry development [5], and institutional pressure [6], as well as internal resource and capability factors such as board faultline [7], board diversity [8, 9], board capital [10], executive types [11], executive team characteristics [12, 13]. Existing studies state that except for the external environment, the team composition of the board and management will have a significant impact on the formulation of enterprise strategy. Still, most of them only emphasize the role of the governance subject at the management level, ignoring the control and values of shareholders at the ownership level, which is the leading force to determine the strategic choice. Especially in SOEs, the state-owned controlling shareholders have a high degree of equity concentration, and only appoint their profit-seeking relationship personnel to the board, resulting in serious agency problems such as bureaucratic control and supervision failure, making the strategic decision of the enterprise follow the government will. It is difficult to reflect the values of heterogeneous governance subjects. New institutional economics believes that the institutional environment may change the internal governance structure of enterprises and then have a significant impact on strategic behavior. Therefore, under the perfection of the enterprise’s internal control structure by the external institution, the governance effects of the shareholder power at the ownership level and the board power at the management level on the enterprise strategic choice will be the focus of this paper.

Since the "Decision of the Central Committee of the Communist Party of China on Some Major Issues Concerning Comprehensively Deepening the Reform" in 2013 proposed vigorously developing a mixed economy, mixed-ownership reform has become a considerable direction for the development of SOEs. The state hopes to transfer part of the equity in the government’s hands to non-state-owned capital strategic investors through mixed-ownership reform to realize the reconfiguration of SOEs’ ownership structure and board composition [14]. It further improves the SOEs’ governance mechanism and revitalizes their development and investment vitality. Thence, mixed-ownership reform has become an important driving force for changing the strategic positioning of SOEs. Different strategic choices have significant distinctions in risk level, investment cycle, income characteristics, etc. The resource base, information channels, interest demands, and risk preferences among heterogeneous shareholders of SOEs are also imparity. It provides an ideal experimental scene for studying how the shareholder power and board power restructuring of heterogeneous shareholders affects the strategic choice in SOEs undergoing mixed-ownership reform.

This paper discusses the impact of mixed-ownership reform on the SOEs’ strategic choice and its mechanism from shareholder power and board power. The main contributions are as follows: (1) It provides a new direction for the governance of non-state-owned shareholders under China’s special institutional background. The existing literatures mainly examine the impact of mixed-ownership reform on the specific behaviors of enterprises [15–19]. This paper concentrates on the strategic choice in strategic management theory, investigates the impact of mixed-ownership reform on the strategic choice of SOEs at the overall level, enriches the relevant literature on the economic consequences of mixed-ownership reform and promotes the cross-domain integration of corporate governance and strategic management. (2) It expands the relevant research on the influencing factors of strategic choice. The external environment, internal resources and capabilities, and enterprise’s strategic choice are long-term concerns in academic circles. By taking the mixed-ownership reform as the starting point, this paper extends the influencing factors of an enterprise’s strategic choice to the control field of the corporate governance subject under the institutional environment, and it also conducts an in-depth analysis of how the dual control rights of non-state-owned shareholders play a role in the strategic choice of SOEs. (3) It furnishes evidence support for the large sample of empirical research to explore the strategic choice of SOEs with mixed-ownership reform. In the past, the problem of internal and external environmental factors and the enterprise’s strategic choice was mainly investigated by analysis and reasoning. We use a large sample empirical method to investigate the impact, mechanism and boundary conditions of mixed-ownership reform on the SOEs’ strategic choice, which provides a useful supplement for the research in this field.

## 2. Theoretical analysis and research hypothesis

The existing management literature involves a variety of enterprise strategy classification frameworks. Among them, the classification framework of Miles et al. [1] is more appropriate to the specific strategies formulated by enterprises in actual operation and management, which has high recognition and wide applicability. According to the division of Miles, Snow [20], the strategic types are divided into Prospector, Analyzer, Reactor, and Defender. Since the reactor strategy mainly follows the mainstream opinions of the industry and reflects the general direction of the current strategic choice in the industry, it is difficult for the strategic activities carried out under this strategic direction to reflect the subjective will of the shareholders and directors within the enterprise. Therefore, this paper removes the reactor, a strategy type that lacks subjective judgment, and adopts prospector strategy, analyzer strategy, and defender strategy to represent the enterprise’s strategic choice. The prospector strategy regards innovation and differentiation as the fundamental way to improve strategic competitiveness, pays attention to product creation, technology innovation, and market development, and can actively seek new strategic opportunities in the uncertain market environment. Still, the risks undertaken and income obtained by the enterprise are highly volatile. The defender strategy focuses on existing products and services to protect against risks and maintain existing market share by reducing production costs, increasing production efficiency, and improving the service quality. The enterprises that choose this strategy have a weak ability to cope with changes in the external environment, face fewer risks, slow performance growth and lack enthusiasm for developing new markets and new businesses. The characteristics of the analyzer strategy are generally intermediate between the above two strategies.

The strategic choice is the evolution result of the mutual game of internal governance participants’ control rights under the guidance of their resource base and interest demands. Therefore, the relative control rights of owners are essential to the strategic choice of enterprises. The new round of mixed-ownership reform guide non-state shareholders to hold shares and appoint directors to participate in corporate governance [21]. On the one hand, it empowers shareholder power of non-state-owned capital, realizes mutual checks and balances among heterogeneous shareholders, establishes a market competition mechanism, and promotes the SOEs’ strategy adjustment [22]. On the other hand, in mixed-ownership reform, non-state-owned shareholders with different characteristics aim to obtain a certain degree of actual control of SOEs and begin to appoint or even over-appoint directors to participate in enterprise operation. At this time, the discretionary power of directors and executives appointed by state-owned shareholders is limited, eventually affecting the enterprise’s strategic decision. Accordingly, starting from the shareholder power and board power of non-state-owned shareholders, this paper explores whether and how the internal power structure of SOEs with mixed-ownership reform affects strategic choices.

### 2.1 Mixed-ownership reform and SOEs’ strategic choice

(1) *Shareholder power level*. Owners of heterogeneous natures often have unique and potentially conflicting preferences in strategic choices [23]. The state-owned controlling shareholders put the policy task first and ensure the stabilization of employment and tax contribution by maintaining the existing products and market share. In contrast, non-state-owned shareholders seek development and income and tend to continuously develop new products and markets to make SOEs occupy a leading position. Before the mixed-ownership reform, public property rights attributes and soft budget constraints made governance problems such as the interest encroachment of state-controlling shareholders common in SOEs [24]. The state’s direct control of SOEs through ownership hinders the implementation of its market-based changes [25]. In the case of a high concentration of equity, the behaviors of SOEs’ managers are less restricted by external investors and supervisors, and they also lack the autonomy of information disclosure and strategic adjustment [26]. It is easy for SOEs to maintain sustained operating activities and strategic behaviors. The longer the ownership structure concentrates on the state-owned controlling shareholders, the more consistent the values of each owner in strategic decisions, and the less sensitive they are to the changes of the external environment, the more likely it is for enterprises to adopt defender strategy.

The ownership structure of SOEs is critical to the impact of the strategic changes. In the process of SOEs’ mixed-ownership reform, it is necessary to reasonably allocate ownership and adjust enterprise strategy with the help of pivotal shareholder power. On one side, SOEs have introduced diversified stakeholders through mixed-ownership reform, which alleviated the internal corporate governance problem and improved SOEs’ efficiency and competitiveness [27]. Some non-state-owned strategic investors have creative thinking and pioneering spirit, attach importance to product development and marketing, and help SOEs become first movers in the market by exploring new products and market opportunities. In terms of information acquisition, the diversified investment experience and professional knowledge of non-state-owned shareholders help them better collect and process information, and be able to timely assess the situation and seize breakthrough market opportunities in the process of strategic selection. On the other side, the higher the shareholding ratio of non-state-owned shareholders, the greater the loss they suffer when the controlling shareholders take opportunistic behaviors. At this time, the non-state-owned shareholders are more active in supervision, which improves the risk-taking level of SOEs [28], and provides conditions for SOEs to choose pioneering and risky strategies. Moreover, the increase in the types and proportions of non-state-owned shareholders has brought equity financing to SOEs. While reducing the financial pressure that enterprises may encounter when choosing a prospector strategy, the strategic resources that controlling shareholders have occupied for a long time have also been released. It provides an opportunity for SOEs to transform into prospectors.

Based on the above analysis, this paper puts forward the following hypotheses:

*H1*: *The higher the diversity of mixed shareholders*, *the greater the tendency of SOEs to choose the prospector strategy*.
*H2*: *The higher the depth of mixed equity*, *the greater the tendency of SOEs to choose the prospector strategy*.

(2) *Board power level*. The appointment of directors by state-owned shareholders is largely subject to government interference; the career experience in SOEs may only be a bargaining chip for their political promotion; they may take short-sighted investments to improve enterprise performance in the short term [29]. The original managers of SOEs to enjoy a "comfortable life", seek "no mistakes" rather than "active merit" [30], try to avoid the high risks and excessive efforts brought by the prospector strategic decision, and finally miss the new market opportunities. In the absence of effective supervision and incentive, management is usually risk-averse when proposing strategic options, the benefits of a high-risk prospector strategy are difficult to achieve during their tenure, and the failures of new market expansion and new product investment will also have a negative impact on their income, appointment, and promotion [31]. Managers and directors of SOEs tend to adopt a sound defender strategy. In addition, directors and managers representing the interests of state-owned controlling shareholders lack attention and understanding of the SOEs’ operating conditions, making it difficult to respond quickly to market changes, limiting the enterprises’ scientific and rational strategic decisions.

The board undertakes to provide advice, approval, and supervision for strategic decisions and communication and coordination with external stakeholders [32]. After the mixed-ownership reform, the internal power structure of the board has changed, which will affect the SOEs’ strategic choice in the following aspects: Firstly, the resource dependence theory points out that power mainly comes from the degree of resource dependence. The appointment of directors by non-state shareholders means that they have invested vital resources in SOEs. The greater the board power of non-state-owned shareholders, the more heterogeneous resources they provide for SOEs, and the higher their voice in approving strategic plans. It can vote in favor of prospector strategies that support the sustainable development of SOEs. Secondly, these directors bring heterogeneous resources such as knowledge composition, social and human capital, and relationship networks into SOEs and provide reliable consultation and suggestions for formulating strategic plans. The rational use of these suggestions and resources provides a rich resource base and targeted direction guidance for the pioneering, innovative, and diversified development of SOEs’ strategies. Thirdly, to solve the current dilemma of being difficult to effectively "voice", non-state-owned shareholders can use their resource advantages as chips to win the proportion of the board seats higher than their shareholding ratio, forming the phenomenon of over-appointing directors. In this case, non-state-owned shareholders have a stronger counterweight to state-owned controlling shareholders in the board decisions and play a role in the appointment and assessment of managers, alleviate the information asymmetry between shareholders and managers, enterprises and market, and increase the energy and resource investment of SOEs in the development projects of markets and products. Finally, the directors appointed by non-state-owned shareholders have flexible operating philosophies and efficient decision styles. Most of them can timely adjust strategies in the complex and changeable market environment, seize the fleeting development opportunities, establish an aggressive enterprise atmosphere, and promote SOEs to choose more pioneering strategies.

Based on the above analysis, this paper proposes the following hypotheses:

*H3*: *The higher the control of the mixed equity*, *the more inclined the SOEs are to choose the prospector strategy*.
*H4*: *The higher the excess control of the mixed equity*, *the more inclined the SOEs are to choose the prospector strategy*.

### 2.2 Balance effect

Given the principal-agent theory, the first-type agency conflict is caused by ownership and management rights separation. The second-type agency conflict is caused by the high concentration of equity in SOEs. These two kinds of agency conflicts may hinder the rational formulation of strategic decisions and the valid implementation of strategic activities and negatively impact SOEs’ long-term development. Therefore, effectively restraining the tunneling behaviors of state-owned controlling shareholders and the opportunistic behaviors of managers in SOEs has become the key to establishing a market-oriented operation mechanism, and the balance effect originates from this.

The balance effect of shareholder power and board power of non-state capital in SOEs with mixed-ownership reform is embodied as follows: Firstly, the introduction of non-state-owned shareholders with high shareholding has achieved the decentralized control of SOEs, played an influential role in supervision and balance for state-owned controlling shareholders and managers, and enabled non-state-owned shareholders to exert substantial influence in strategic decisions [33]. The balance of non-state-owned shareholders can significantly reduce the self-interest behaviors of controlling shareholders, such as capital occupation and connected transactions [34, 35]. If the proposals of the controlling shareholders or the management damage the interests of the enterprise, the non-state-owned shareholders can also reach a consensus at the shareholders’ meeting to take action against the controlling shareholders, effectively curbing the excessive speculation of the state-owned controlling shareholders [36, 37], and promote SOEs to choose prospector strategy. Secondly, directors represent the interests of shareholders and play a role in supervising and managing the managers’ behaviors and decisions. When the power of the directors appointed by the state-owned controlling shareholders is too large and in a strong position, it is easy to monopolize the board, resulting in the imbalance of SOEs’ power distribution, which may seriously weaken the internal supervision. The appointment of directors by non-state-owned shareholders weakens the influence of controlling shareholders in the board arrangement [38], reduces management’s inefficient behaviors, and provides rich financial redundancy for SOEs to invest in new fields. Thirdly, non-state-owned shareholders effectively negotiate with state-owned controlling shareholders and managers by control rights to promote SOEs to choose appropriate strategies to increase enterprise wealth. It can be seen that the effective balance of dual control rights between non-state-owned shareholders and state-owned controlling shareholders can affect the enterprise strategic choice.

According to the above analysis, this paper proposes the following hypothesis:

*H5*: *Mixed-ownership reform can influence SOEs’ strategic choice through the balance effect between heterogeneous shareholders with the same power*.

### 2.3 Synergy effect

In the context of the relatively concentrated equity of SOEs, the interaction between the shareholder power and the board power is difficult to be separated. The impact of ownership structure and board composition on enterprises’ strategic decisions is also closely related [39]. Zhang et al. [40] pointed out that shareholder power and board power on strategic choices are not independent in the long-term development of SOEs. When the resources of shareholder power support the board power, it can significantly promote the SOEs’ strategic adjustment. During the mixed-ownership reform process, if SOEs can give non-state-owned shareholders sufficient board seats to encourage them to provide resource support, the non-state-owned capital’s shareholder power and board power can cooperate to help enterprises make scientific strategic decisions, and the synergy originates from this. In theory, SOEs adopt the "one share, one vote" voting method, and non-state-owned shareholders can elect directors through cumulative voting and obtain board seats that match their shareholding ratios. However, this is not the case in reality. Non-state shareholders rarely get board seats commensurate with their shareholder power. Many non-state-owned shareholders fail to appoint directors in SOEs or obtain board seats that are lower than their shareholding ratios, making it difficult for such non-state-owned shareholders to participate in the governance of SOEs, lack the willingness of resource support for the board’s strategic decisions, and form resource constraints on the board power of other non-state-owned shareholders. Ultimately, the board power of non-state-owned shareholders in SOEs fails to coordinate with shareholder power to influence strategic decisions effectively.

However, some SOEs have a special situation: non-state-owned shareholders can over-appoint directors. Baidu, Alibaba, Tencent, and JD, the non-state strategic investors introduced by China Unicom during the mixed-ownership reform, held 3.3%, 2.04%, 5.18%, and 2.36% of the shares, respectively. Still, each appointed one director on the board. It far exceeds the number of directors that can be appointed by its shareholding ratio, which enables all non-state-owned shareholders to jointly veto strategic decisions that are unfavorable to the enterprises’ sustainable development in the board vote, protecting the decision-making power of strategic investors [41]. Therefore, if the non-state-owned shareholders are given the power to over-appoint directors, they will take shares to obtain the strategic decision-making power. The directors who can effectively voice are willing to negotiate to achieve a consistent strategy, balance the interest demands of each non-state-owned shareholder and obtain their resource support, resulting in a virtuous circle of mutual promotion between the shareholder power and the board power. It allows SOEs to utilize the advantages of non-state-owned capital more fully and open up new development spaces. It can be seen that although the board power of non-state-owned capital can impact the strategy of SOEs. If the proportion of directors they obtained is less than the shareholding proportion, it is challenging to play a role in the strategy choice in coordination with shareholder power. When non-state-owned shareholders over-appoint directors, each non-state-owned shareholder has the opportunity to express their opinions in the decision-making of the board. It is willing to supervise the daily operation of enterprises, provide rapid and effective resource support, and maximize the governance effect of the board power.

Based on the above analysis, this paper proposes the following hypothesis:

*H6*: *In the case of non-state shareholders over-appointing directors*, *mixed-ownership reform can influence SOEs’ strategic choice through the synergy effect of non-state shareholders’ shareholder power and board power*.

### 3. Materials and methods

### 3.1. Data

The share-trading reform was basically completed at the end of 2007. After that, it became a common phenomenon for non-state-owned strategic investors to take shares in SOEs. Therefore, this paper selects China’s Shanghai and Shenzhen A-share state-owned listed companies from 2008 to 2019 as the research objects. Since there is a time lag in the impact of mixed-ownership reform on SOEs’ strategies, the sample interval of the enterprise strategy is forwarded by one-period from 2009 to 2019, and explanatory variables and control variables are the current period from 2008 to 2018. It can alleviate the endogeneity problem caused by reverse causality. In line with the needs of the research questions, the samples are eliminated as follows: (1) ST, *ST, and financial industry samples (ST represents a stock that has received a special treatment due to two consecutive years of losses in the domestic listed company; *ST represents a stock that have been warned of delisting risk due to three consecutive years of losses in the domestic listed company); (2) data anomaly (asset-liability ratio greater than 1 or operating income less than 0) samples; (3) samples with missing main variables; (4) samples with the nature of shareholders cannot be determined from databases, annual reports, and the companies’ website, leading ultimately to 7 976 firm-year observations. Moreover, as there are new listed, withdrawn and completely privatized SOEs every year, the sample of eligible state-owned listed companies is not the same from year to year. There were 672 samples in 2008, 678 samples in 2009, 669 samples in 2010, 666 samples in 2011, 712 samples in 2012, 740 samples in 2013, 729 samples in 2014, 751 samples in 2015, 773 samples in 2016, 772 samples in 2017, and 814 samples in 2018.

Among them, the basic data of mixed-ownership reform is mainly obtained by manually sorting out the nature, shareholding ratio, shareholder relations, and appointed directors of the top ten shareholders disclosed in the annual reports and relevant information websites of state-owned listed companies. In addition, the industry classification comes from the WIND database, the data for other variables come from the CSMAR database and the CNRDS database, and STATA.14.0 is used for data processing and empirical analysis. All continuous variables are winsorized at 1% and 99% to avoid the influence of extreme values on the research results.

### 3.2. Major variable construction

#### 3.2.1. Explained variable

The explained variable is enterprise strategy (*SP*). We draw on the index construction method of enterprise strategy in the research of Bentley et al. [42], Higgins et al. [43], Bentley-Goode et al. [44], and Xing et al. [45], and measures from the following six aspects of enterprise characteristics:

(1) Propensity to develop new products (intangible assets/sales revenue). Prospectors are more likely to develop new products and enter new markets than defenders, and this ratio is higher. (2) Production and operation efficiency (number of employees/sales revenue). The larger the ratio, the lower the efficiency of the enterprise. Prospector strategy focuses on the long-term development of enterprises, pays less attention to organizational efficiency, and weak unit employees’ ability to generate income. (3) Company growth (sales revenue growth rate). Prospectors keep making progress, which makes them have a faster growth rate. Defenders are more conservative in the development process, and their growth rate is relatively slow. (4) Product market development tendency ((selling expenses + administrative expenses)/sales revenue). The larger the ratio, the higher the expenditure on administration and marketing. Compared with the defender strategy, the prospector strategy places more importance on product promotion and market development, and the administrative and marketing expenses are higher. (5) Organizational stability (standard deviation of employee number over the past five years/average employee number). Due to the continuous changes in product and business markets, prospector enterprises constantly update their employees, which leads to great volatility of employees. (6) Capital intensity (fixed assets/total assets). The larger the index, the higher the degree of enterprises using machinery equipment rather than labor. Prospector enterprises emphasize organizational flexibility, reduce the degree of standardization by strengthening labor’s role and have low capital intensity.

For the above six indicators, this paper measures according to their rolling five-year average and divides them into five groups from small to large in each year-industry sample. The first five are positive indicators, the smallest group is assigned a value of 1, the second group is assigned a value of 2, and so on, the largest group is assigned a value of 5, the sixth is a negative indicator, the largest group is assigned a value of 1. The second largest group is assigned a value of 2, and so on, the smallest group is assigned a value of 5. Then, sum up the corresponding values of the six indicators of all enterprises, and finally obtain the enterprise strategy index (*SP*) with a value range of 6–30. The larger the value of *SP*, the more likely the enterprise is to adopt a prospector strategy. On the contrary, the smaller the value of *SP*, the more likely the enterprise is to adopt a defender strategy.

#### 3.2.2. Explanatory variables

The explanatory variable is the degree of SOEs’ mixed-ownership reform (*MIX*), mainly measured from the reconfiguration of shareholder power and board power. The dimension of shareholder power mainly considers the number and shareholding proportion of heterogeneous shareholders among the top ten shareholders of SOEs, including the diversity of mixed shareholders (*MIXS*) and the depth of mixed equity (*MIXO*). The dimension of board power mainly considers the appointment and over-appointment of directors by non-state-owned shareholders in SOEs, including the control of mixed equity (*NONSOE_D*) and the excess control of mixed equity (*NONSOE_OD*).

The diversity of mixed shareholders (*MIXS*), drawing on the research of Wang, Song [46], the Herfindahl index of shareholder categories (*HHI* = 1−∑*p*_*i*_^2^, *P*_*i*_ represents the proportion of the class *i* shareholders in the top ten shareholders) is used to measure. The depth of mixed equity (*MIXO*), with reference to the practice of Ma et al. [47], the sum of the shareholding proportion of the four non-state-owned shareholders among the top ten shareholders, namely private, foreign, natural and institutional investors, is used to measure. The control of mixed equity (*NONSOE_D*) is defined as the proportion of directors appointed by non-state-owned shareholders among the top ten shareholders [48]. The excess control of mixed equity (*NONSOE_OD*) is defined as the proportion of over-appointed directors by non-state-owned shareholders among the top ten shareholders[49].

#### 3.2.3. Control variables

In order to control many other factors that may affect the strategic choice of SOEs, this paper uses the research of Zeng et al. [50], Meng et al. [4] and Shang et al. [51] for reference to control from the following three aspects. In terms of enterprises’ basic characteristics, six variables are selected: company size (*SIZE*), asset-liability ratio (*LEV*), profitability (*TQ*), cash flow ratio (*CF*), financial distress (*Z_score*), and tangible assets ratio (*TA*). In the aspect of enterprise governance characteristics, there are separation of two rights (*TS*), board size (*BOARD*), and the proportion of independent directors (*INDEP*) three variables. The accounting firm scale (*BIG4*) is controlled in terms of audit quality characteristics. Moreover, the market environment and economic development level of the region where the enterprise is located may also have an impact on the SOEs’ strategic choice, controlling the marketization degree (*MAR*) of the province where the enterprise is registered. The specific variable definitions and descriptions are shown in Table 1.

**Table 1:** The variable definition and description.

Variable type	Variable name	Symbol	Measurement of variable	Reference
Explained variable	enterprise strategy	*SP*	The discrete variable constructed by the scores of the above six indicators is used to measure enterprise strategy	Bentley et al. [42], Higgins et al. [43], Bentley-Goode et al. [44], and Xing et al. [45]
Explanatory variable	The diversity of mixed shareholders	*MIXS*	Herfindahl index of shareholder categories *HHI* = 1−∑*p*_*i*_^2^, *P*_*i*_ represents the proportion of the class *i* shareholders in the top ten shareholders	Wang, Song [46]
	The depth of mixed equity	*MIXO*	The sum of the four non-state-owned shareholding ratios of private, foreign, natural persons, and institutional investors in the top ten shareholders	Ma et al. [47]
	The control of mixed equity	*NONSOE_D*	The proportion of directors appointed by non-state-owned shareholders among the top ten shareholders	Ma et al. [48]
	The excess control of mixed equity	*NONSOE_OD*	(Board seats appointed by non-state shareholders − board seats that non-state-owned shareholders should obtain)/total number of directors	Li et al. [49]
Control variable	Company size	*SIZE*	Natural logarithm of the total market value of listed companies	Yuan et al. [52]
	Asset-liability ratio	*LEV*	Total liabilities / total assets	Zhang et al. [40], Shang et al. [51]
	Profitability	*TQ*	The company’s market value/asset replacement cost	Li et al. [49]
	Cash flow ratio	*CF*	Net cash flow from operating activities/total assets	Zeng et al. [50]
	Financial distress	*Z_score*	1.2×working capital/total assets+1.4×retained earnings/total assets+3.3×earnings before interest and tax/total assets+0.6×total market value /total liabilities+0.9996×operating income/total assets	Zhang et al. [40]
	Tangible assets ratio	*TA*	(Tangible fixed assets + inventory)/total assets	Xu et al. [53]
	Separation of two rights	*TS*	Difference between control and ownership	Li et al. [49]
	Board size	*BOARD*	Natural logarithm of the number of directors on the board	Zeng et al. [50]
	The proportion of independent directors	*INDEP*	The number of independent directors/the number of directors on the board	Meng et al. [4]
	Accounting firms scale	*BIG4*	If the audit unit is the "Big four accounting firms", it takes 1; otherwise, it takes 0	Sun et al. [54]
	The marketization degree	*MAR*	If the company is registered in the eastern region, the value is 1; otherwise, the value is 0	Meng et al. [4]

### 3.3. Multiple regression model

Drawing on the research of Yang et al. [55], this paper establishes model (1) to test the impact of mixed-ownership reform on the strategic choice of SOEs:

$${SP}_{i,t+1}=\alpha_{0}+\alpha_{1}{MIX}_{i,t}+\alpha_{2}{CONTROLS}_{i,t}+\eta_{YEAR}+\eta_{IND}+\varepsilon_{i,t}$$ (1)

Where *i* and *t* represent the firm and year, and *ε* is the residual of the regression model. The explained variable *SP*_*i*,*t+1*_ delegates the enterprise strategy of firm *i* in year *t*+1. The explanatory variable is the degree of mixed-ownership reform in SOEs. *CONTROLS* is a batch of control variables. *η*_*YEAR*_ and *η*_*IND*_ refer to the fixed effects at the year and industry levels, respectively, which can effectively alleviate the endogenous problems caused by missing variables. We adopted T-statistics adjusted for the robust standard error of clustering at the firm level to alleviate the possible sequence-related problems in all models to ensure the research conclusions’ robustness.

## 4. Results

### 4.1. Summary statistics

Table 2 presents the descriptive statistical results of the main variables. The mean value of enterprise strategy (*SP*) is 18.040, and the standard deviation is 3.844, indicating certain disparities in strategic types among different state-owned listed companies, with sufficient variability. This is similar to the statistical results of Meng et al. [56] and Wang et al. [57], using all listed companies as samples. In the shareholder power dimension, the mean value of the diversity of mixed shareholders (*MIXS*) is 0.267, stating that the diversification of heterogeneous shareholders in SOEs needs to be improved. The mean value of the depth of mixed equity (*MIXO*) is 0.102, manifesting that the average shareholding ratio of non-state-owned shareholders is 10.2%, which is lower than the mean value of the state-owned shareholding ratio of 46.7%, and the problem of "One big share alone" in SOEs has not been effectively solved. In the board power dimension, the mean value of the control of mixed equity (*NONSOE_D*) is 0.021, indicating that the average proportion of directors appointed by non-state-owned shareholders in SOEs is 2.1%, which is much lower than the average shareholding ratio of non-state-owned shareholders. The mean value of the excess control of mixed equity (*NONSOE_OD*) is -0.099, the median is -0.070, and the maximum value is 0.319, illustrating that only a few non-state-owned shareholders of SOEs have over-appointed directors. Still, the largest proportion of over-appointed directors reaches 31.9%, reflecting the higher level of over-appointed directors among SOEs in which non-state-owned shareholders have obtained excess board seats.

**Table 2:** Descriptive statistics of the main variables.

Variable	N	Mean	Std	Min	P50	P25	P75	Max
*SP*	7976	18.040	3.844	6.000	18.000	15.000	21.000	30.000
*MIXS*	7976	0.267	0.165	0.020	0.229	0.128	0.397	0.676
*MIXO*	7976	0.102	0.092	0.006	0.069	0.035	0.135	0.461
*NONSOE_D*	7976	0.021	0.061	0.000	0.000	0.000	0.000	0.444
*NONSOE_OD*	7976	-0.099	0.126	-0.558	-0.070	-0.148	-0.030	0.319
*STATE*	7976	0.467	0.151	0.113	0.470	0.353	0.570	0.855
*SIZE*	7976	22.690	1.098	19.850	22.540	21.910	23.350	26.560
*LEV*	7976	0.517	0.194	0.075	0.529	0.374	0.666	0.937
*TQ*	7976	1.809	1.091	0.785	1.438	1.129	2.050	9.451
*CF*	7976	0.050	0.071	-0.187	0.048	0.010	0.091	0.339
*Z_score*	7976	3.852	4.251	-0.215	2.544	1.508	4.432	39.32
*TA*	7976	0.372	0.201	0.014	0.221	0.353	0.513	0.827
*TS*	7976	0.041	0.075	0.000	0.000	0.000	0.041	0.299
*BOARD*	7976	2.216	0.197	1.609	2.197	2.197	2.303	2.708
*INDEP*	7976	0.368	0.053	0.250	0.333	0.333	0.375	0.600
*BIG4*	7976	0.091	0.288	0.000	0.000	0.000	0.000	1.000
*MAR*	7976	0.584	0.493	0.000	1.000	0.000	1.000	1.000

In addition, according to the practice of Ma et al. [48], this paper makes statistics on P50 (median), P25 (25th quantile) and P75 (75th quantile) of the main variables. It is found that the number of SOEs with non-state-owned shareholders appointing and over-appointing directors is relatively small. The board power of non-state-owned shareholders needs to be further improved in SOEs. The statistical results of other control variables are basically consistent with the existing research [16, 40].

### 4.2. Baseline results

Table 3 reports the regression results of the impact of mixed-ownership reform on the strategic choice of SOEs. Column (1) shows that the diversity of mixed shareholders (*MIXS*) is significantly positively correlated with enterprise strategy (*SP*) (*α*_1_ = 2.301, *P*<0.01), indicating that with the improvement of the degree of shareholder diversity, SOEs continue to make strategic adjustments in the direction of prospectors and seek breakthrough development. Column (2) shows a significant positive correlation between the depth of mixed equity (*MIXO*) and enterprise strategy (*SP*) (*α*_1_ = 2.534, *P*<0.01), declaring that the higher the shareholding ratio of non-state-owned shareholders, the more able to promote SOEs to carry out prospector strategies. Column (3) displays that the control of mixed equity (*NONSOE_D*) has a significant positive correlation with enterprise strategy (SP) (*α*_1_ = 3.894, *P*<0.01), which states that the higher the proportion of board seats obtained by non-state-owned shareholders, the more likely it is for the board and management to make strategic decisions that converge with the interests of non-state-owned shareholders. Column (4) demonstrates that there is also a significant positive correlation between the excess control of mixed equity (*NONSOE_OD*) and enterprise strategy (*SP*) (*α*_1_ = 5.185, *P*<0.01), which manifests that the higher the proportion of directors over-appointed by non-state-owned shareholders, the greater the possibility of SOEs choosing prospector strategies. From the above results, it can be seen that hypotheses H1-H4 are verified.

**Table 3:** The impact of mixed-ownership reform on the strategy choice of SOEs.

*Y = SP*	(1)	(2)	(3)	(4)
*MIXS*	2.301***			
	(4.40)			
*MIXO*		2.534**		
		(2.42)		
*NONSOE_D*			3.894***	
			(3.15)	
*NONSOE_OD*				5.185***
				(2.75)
*SIZE*	-0.037	-0.047	0.009	0.008
	(-0.34)	(-0.43)	(0.08)	(0.07)
*LEV*	-10.780***	-10.880***	-10.870***	-10.900***
	(-9.53)	(-9.55)	(-9.60)	(-9.62)
*TQ*	0.817***	0.840***	0.836***	0.841***
	(8.38)	(8.60)	(8.62)	(8.67)
*CF*	-3.363***	-3.323***	-3.298***	-3.259***
	(-3.59)	(-3.53)	(-3.50)	(-3.46)
*Z_score*	-0.087**	-0.089**	-0.089**	-0.090**
	(-2.24)	(-2.30)	(-2.34)	(-2.35)
*TA*	-12.990***	-13.080***	-13.180***	-13.190***
	(-9.76)	(-9.75)	(-9.89)	(-9.89)
*TS*	-0.384	-0.715	-0.682	-0.707
	(-0.29)	(-0.54)	(-0.51)	(-0.53)
*BOARD*	-0.320	-0.335	-0.357	-0.340
	(-0.62)	(-0.64)	(-0.69)	(-0.66)
*INDEP*	0.164	0.161	0.325	0.217
	(0.11)	(0.10)	(0.21)	(0.14)
*BIG4*	-1.448***	-1.473***	-1.375***	-1.380***
	(-4.07)	(-4.14)	(-3.92)	(-3.93)
*MAR*	-0.262	-0.269	-0.307	-0.293
	(-1.17)	(-1.20)	(-1.37)	(-1.30)
*CONSTANT*	27.72***	28.41***	27.36***	27.42***
	(10.36)	(10.51)	(10.25)	(10.25)
*YEAR F*.*E*.	*YES*	*YES*	*YES*	*YES*
*IND F*.*E*.	*YES*	*YES*	*YES*	*YES*
*N*	7976	7976	7976	7976
*adj*. *R*^*2*^	0.148	0.142	0.142	0.141

Note: The t-statistics are reported in parentheses on robust standard errors clustered at the firm level

*, ** and *** designate statistical significance at the 10%, 5%, and 1% level, respectively.

From the control variables, the asset-liability ratio (*LEV*), financial distress (*Z_score*), cash flow ratio (*CF*) and tangible assets ratio (*TA*) are significantly negatively correlated with the enterprise strategy, indicating that when the enterprise debt level is high, SOEs tend to adopt defender strategy to pursue stability. When the proportion of tangible assets is high, it means that SOEs have purchased a large number of machinery and equipment for standardized production, and are more willing to carry out the defender strategy. The cash flow generated from operating activities may continue to be used for the original operating activities, thereby inhibiting the SOEs from adjusting to the prospector strategy. Profitability is significantly positively correlated with enterprise strategy, which states that the improvement of market value drives SOEs to become prospectors, gains their leading position in the industry, and promotes enterprises to obtain long-term benefits.

The existing studies point out that the mixed-ownership reform can promote the SOEs’ strategic change [40], improve the M&A efficiency [17], and increase the strategic flexibility. On this basis, this study deeply explores the impact of mixed-ownership reform on the SOEs’ strategic choice, and finds that SOEs begin to weaken the defense mechanism to maintain the status quo, and develop into the prospector strategies, which confirms that the strategic flexibility of SOEs is strengthened after the mixed-ownership reform. Meanwhile, it determines the specific strategic adjustment direction of SOEs, providing a useful supplement for the research of enterprise strategic management.

### 4.3. Robustness checks

**4.3.1. Endogenous test.** This paper uses instrumental variable analysis, propensity score matching, and multiple-period forwarded explained variable to deal with endogenous problems.

*1*. *Instrumental variable analysis*. Given some unobservable factors that may affect the degree of SOEs’ mixed-ownership reform and the strategy choice in the model (1), there may be endogenous problems caused by missing variables. In the light of Fan et al. [58] and Tang et al. [59], this paper selects the number of coastal ports in each province (*SEA_PORT*) and the unemployment rate in each province (*UNEMP*) as the instrumental variables for the degree of SOEs’ mixed-ownership reform and uses the two-stage least squares method (IV-2SLS) for regression. Table 4 reports the two-stage regression results. Columns (1)-(4) are the regression results of the first stage. The regression coefficient of *SEA_PORT* is significantly positive, indicating that the more ports in the province, the better the marketization degree and economic development level of the region and the higher the degree of non-state-owned shareholders participating in the SOEs’ governance. The second stage regression results in columns (5)-(8) further show that the mixed-ownership reform of SOEs can indeed promote the adjustment of enterprises to prospector strategy under controlling endogenous problems.

**Table 4:** The regression results of two-stage 2SLS.

	First stage				Second stage			
	(1)	(2)	(3)	(4)	(5)	(6)	(7)	(8)
	*MIXS*	*MIXO*	*NONSOE_D*	*NONSOE_OD*	*SP*	*SP*	*SP*	*SP*
*SEA_PORT*	0.003***	0.001***	0.001***	0.001**				
	(9.48)	(6.56)	(5.07)	(2.25)				
*UNEMP*	-0.003	-0.003**	-0.002*	-0.005**				
	(-1.37)	(-2.00)	(-1.68)	(-2.47)				
*MIXS*					21.700***			
					(7.15)			
*MIXO*						57.580***		
						(5.90)		
*NONSOE_D*							96.840***	
							(4.94)	
*NONSOE_OD*								80.950***
								(3.19)
*CONSTANT*	-0.193***	-0.354***	0.033	-0.113***	32.940***	48.270***	24.680***	38.350***
	(-3.65)	(-11.37)	(1.54)	(-2.75)	(20.04)	(11.59)	(10.58)	(8.12)
*CONTROLS*	*YES*	*YES*	*YES*	*YES*	*YES*	*YES*	*YES*	*YES*
*IND/YEAR*	*YES*	*YES*	*YES*	*YES*	*YES*	*YES*	*YES*	*YES*
*N*	7976	7976	7976	7976	7976	7976	7976	7976
*adj*. *R*^*2*^	0.086	0.113	0.038	0.020	—	—	—	—
Hansen test	—	—	—	—	2.404 (p = 0.121)	0.896 (p = 0.344)	0.887 (P = 0.346)	1.660 (P = 0.198)

Note: *, ** and *** designate statistical significance at the 10%, 5%, and 1% level, respectively.

*2*. *Propensity score matching*. We draw on the practice of Ma et al. [48] to alleviate the endogeneity problem caused by the sample selection and use the propensity score matching (PSM) method for regression. We select paired variables that may affect the degree of SOEs’ mixed-ownership reform, group the samples based on the average shareholding proportion of non-state-owned shareholders, and divide the whole sample into treatment group (a group with a higher degree of mixed-ownership reform) and control group (a group with a lower degree of mixed-ownership reform). The nearest neighbor matching method with calipers of 0.05 and put back is used for 1:3 propensity score matching to find the control group sample with the closest propensity score for each company in the treatment group and perform regression based on paired samples.

**Fig 1:**
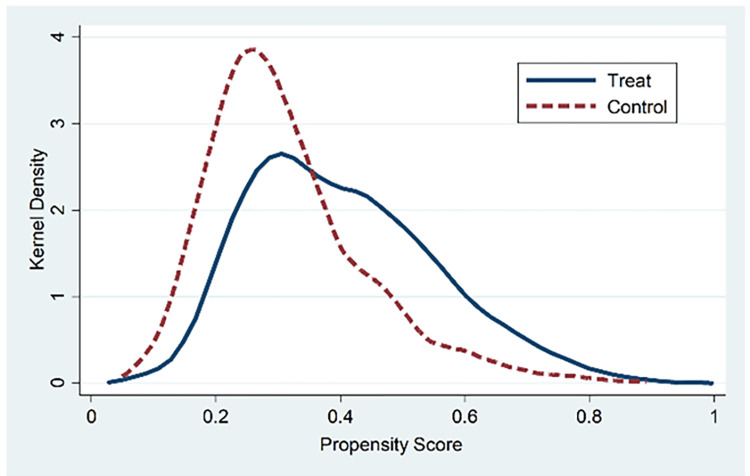
Before matching.

**Fig 2:**
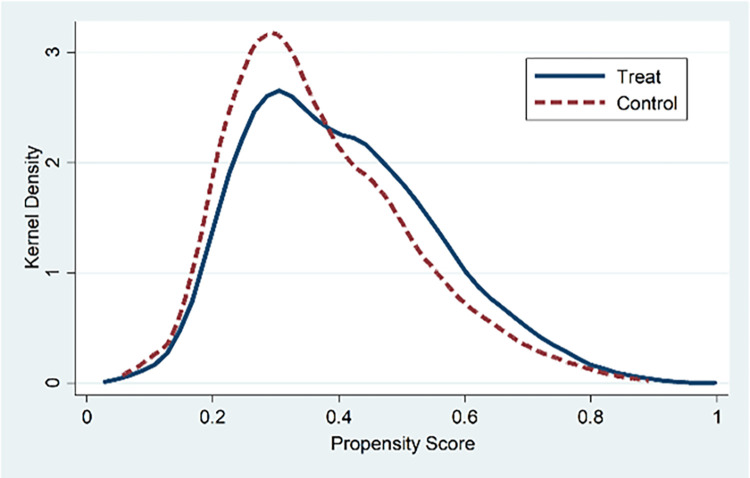
After matching.

Figs 1 and 2 report the distribution of propensity scores before and after matching. It can be found that the probability density of propensity scores of the treatment group and control group is closer than that before matching, indicating that the matching effect of samples is better. Table 5 shows the empirical results after pairing. The diversity of mixed shareholders, the depth of mixed equity, the control of mixed equity, and the excess control of mixed equity are significantly positively correlated with the enterprise strategy. The above results are consistent with the main regression results, which proves the robustness of the research conclusions of this paper.

**Table 5:** The regression results after PSM matching.

*Y = SP*	(1)	(2)	(3)	(4)
*MIXS*	2.462***			
	(4.57)			
*MIXO*		2.608**		
		(2.48)		
*NONSOE_D*			3.619***	
			(2.87)	
*NONSOE_OD*				1.023**
				(2.22)
*CONSTANT*	27.07***	28.02***	27.01***	27.46***
	(10.09)	(10.31)	(10.06)	(10.16)
*CONTROLS*	*YES*	*YES*	*YES*	*YES*
*YEAR F*.*E*.	*YES*	*YES*	*YES*	*YES*
*IND F*.*E*.	*YES*	*YES*	*YES*	*YES*
*N*	6216	6216	6216	6216
*adj*. *R*^*2*^	0.174	0.166	0.166	0.163

Note: The t-statistics are reported in parentheses on robust standard errors clustered at the firm level.

*, ** and *** designate statistical significance at the 10%, 5%, and 1% level, respectively.

*3*. *Multiple-period forwarded explained variable*. Mixed-ownership reform exerts an impact on the SOEs’ strategic choices. Once the strategic positioning of SOEs is determined, non-state-owned strategic investors may be introduced to participate in operating governance according to strategy implementation needs. Therefore, there is a reverse causal problem. Following Fang et al. [60], this part makes the forwarding 2–3 stages of the explained variable. Table 6 lists the test results of enterprise strategy in multiple-period forwarded. The regression coefficients of the diversity of mixed shareholders, the depth of mixed equity, the control of mixed equity, and the excess control of mixed equity are significantly positive, and the previous research conclusions remain unchanged.

**Table 6:** The regression results of multiple-period forwarded explained variable.

	*Forward2(SP2)*				*Forward3(SP3)*			
	(1)	(2)	(3)	(4)	(5)	(6)	(7)	(8)
*MIXS*	2.311***				2.311***			
	(4.13)				(3.98)			
*MIXO*		2.659**				2.986**		
		(2.36)				(2.54)		
*NONSOE_D*			3.536***				3.096**	
			(2.64)				(2.29)	
*NONSOE_OD*				0.990**				1.211**
				(2.16)				(2.54)
*CONSTANT*	31.78***	32.53***	31.49***	31.73***	35.46***	36.27***	35.20***	35.39***
	(10.90)	(11.01)	(10.82)	(10.88)	(11.99)	(12.13)	(11.89)	(11.95)
*CONTROLS*	*YES*	*YES*	*YES*	*YES*	*YES*	*YES*	*YES*	*YES*
*YEAR F*.*E*.	*YES*	*YES*	*YES*	*YES*	*YES*	*YES*	*YES*	*YES*
*IND F*.*E*.	*YES*	*YES*	*YES*	*YES*	*YES*	*YES*	*YES*	*YES*
*N*	6129	6129	6129	6129	5271	5271	5271	5271
*adj*. *R*^*2*^	0.165	0.159	0.159	0.157	0.182	0.177	0.175	0.174

Note: The t-statistics are reported in parentheses on robust standard errors clustered at the firm level.

*, ** and *** designate statistical significance at the 10%, 5%, and 1% level, respectively.

#### 4.3.2. Other robustness tests

This study conducts robustness analyses based on alternative measures of explained and explanatory variables, and alternative model specifications.

*1*. *Alternative measures of explained variable*. We have replaced the measurement method of enterprise strategy to exclude the possible influence of the indicator selection on the research conclusions. Based on the research of Sun et al. [54], the dummy variables of prospector strategy (*PROS*) and defender strategy (*DEFE*) are used to measure the enterprise strategy. If *SP*≥24, it is defined as the prospector strategy, and the value of *PROS* is 1; otherwise, the value is 0. If *SP*≤12, it is defined as the defender strategy, and the value of *DEFE* is 1; otherwise, the value is 0. Table 7 reports the test results of altering the measurement method of enterprise strategy. The diversity of mixed shareholders, the depth of mixed equity, the control of mixed equity, and the excess control of mixed equity have a significant positive correlation with the prospector strategy and a significant negative correlation with the defender strategy. The research conclusions remain stable.

**Table 7:** Alternative measures of explained variable.

	(1)	(2)	(3)	(4)	(5)	(6)	(7)	(8)
	*PROS*	*PROS*	*PROS*	*PROS*	*DEFE*	*DEFE*	*DEFE*	*DEFE*
*MIXS*	0.189***				-0.133***			
	(3.22)				(-3.59)			
*MIXO*		0.259**				-0.100		
		(2.30)				(-1.28)		
*NONSOE_D*			0.389***				-0.259***	
			(2.59)				(-4.13)	
*NONSOE_OD*				0.110**				-0.049**
				(2.15)				(-1.98)
*CONSTANT*	1.114***	1.189***	1.082***	1.110***	-0.369**	-0.392**	-0.347*	-0.362**
	(3.83)	(4.05)	(3.73)	(3.84)	(-2.02)	(-2.11)	(-1.91)	(-1.98)
*CONTROLS*	*YES*	*YES*	*YES*	*YES*	*YES*	*YES*	*YES*	*YES*
*YEAR F*.*E*.	*YES*	*YES*	*YES*	*YES*	*YES*	*YES*	*YES*	*YES*
*IND F*.*E*.	*YES*	*YES*	*YES*	*YES*	*YES*	*YES*	*YES*	*YES*
*N*	7976	7976	7976	7976	7976	7976	7976	7976
*adj*. *R*^*2*^	0.092	0.090	0.090	0.089	0.048	0.043	0.045	0.043

Note: The t-statistics are reported in parentheses on robust standard errors clustered at the firm level.

*, ** and *** designate statistical significance at the 10%, 5%, and 1% level, respectively.

*2*. *Alternative measures of explanatory variables*. According to the research of Zhu et al. [61], the entropy index (*EI* = ∑*Q*_*j*_×ln (1/*Q*_*j*_), *Q*_*j*_ represents the proportion of shares held by class *j* shareholders in the total number of shares held by the top ten shareholders) is used to measure the depth of mixed equity (*MIXO1*). The control of mixed equity (*DUM_D*) is measured by whether non-state-owned shareholders appoint directors. The excess control of mixed equity (*DUM_OD*) is measured by whether non-state-owned shareholders have over-appointed directors. The test results are shown in Table 8. The depth of mixed equity, the control of mixed equity, and the excess control of mixed equity are significantly positively correlated with enterprise strategy. The results are consistent with the previous one.

**Table 8:** Alternative measures of explanatory variables.

*Y = SP*	(1)	(2)	(3)
*MIXO1*	1.557***		
	(4.82)		
*DUM_D*		0.659***	
		(2.81)	
*DUM_OD*			0.711***
			(2.89)
*CONSTANT*	27.250***	27.370***	27.400***
	(10.23)	(10.24)	(10.24)
*CONTROLS*	*YES*	*YES*	*YES*
*YEAR F*.*E*.	*YES*	*YES*	*YES*
*IND F*.*E*.	*YES*	*YES*	*YES*
*N*	7976	7976	7976
*adj*. *R*^*2*^	0.149	0.142	0.141

Note: The t-statistics are reported in parentheses on robust standard errors clustered at the firm level.

*, ** and *** designate statistical significance at the 10%, 5%, and 1% level, respectively.

*3*. *Robustness test based on a negative binomial regression model*. Since the value of enterprise strategy is a non-negative integer, which is a typical count variable, the negative binomial regression (*NBreg*) is used for estimation proposed by Meng et al. [4]. The regression results are shown in Table 9. There is a significant positive correlation between the diversity of mixed shareholders, the depth of mixed equity, the control of mixed equity, and the excess control of mixed equity and the enterprise strategy, and the results remain stable.

**Table 9:** Alternative empirical model.

*Y = SP*	(1)	(2)	(3)	(4)
*MIXS*	0.127***			
	(7.73)			
*MIXO*		0.138***		
		(4.55)		
*NONSOE_D*			0.212***	
			(4.90)	
*NONSOE_OD*				0.0541**
				(2.54)
*CONSTANT*	3.413***	3.446***	3.393***	3.407***
	(45.35)	(45.39)	(45.11)	(45.26)
*CONTROLS*	*YES*	*YES*	*YES*	*YES*
*IND/YEAR*	*YES*	*YES*	*YES*	*YES*
*N*	7976	7976	7976	7976
*Pseudo R* ^*2*^	0.022	0.021	0.021	0.020

Note: The t-statistics are reported in parentheses on robust standard errors clustered at the firm level.

*, ** and *** designate statistical significance at the 10%, 5%, and 1% level, respectively.

### 4.4. Mechanism test results

Based on the test results of baseline regression, it can be seen that the shareholder power and board power of non-state-owned capital positively impact the choice of prospector strategies in SOEs. Then, the following research focuses on the mechanism of mixed-ownership reform affecting the SOEs’ strategic choice.

#### 4.4.1. Balance effect

Drawing on the research of Li et al. [62], we construct models (2) and (3) to test whether the shareholder power and board power of non-state-owned capital in SOEs with mixed-ownership reform have a balance effect on state-owned controlling shareholders and then affect enterprises’ strategic choice.


$${SP}_{i,t+1}=\alpha_{0}+\alpha_{1}{SPB}_{i,t}+\alpha_{2}{CONTROLS}_{i,t}+\eta_{YEAR}+\eta_{IND}+\varepsilon_{i,t}$$ (2)

$${SP}_{i,t+1}=\alpha_{0}+\alpha_{1}{DPB}_{i,t}+\alpha_{2}{CONTROLS}_{i,t}+\eta_{YEAR}+\eta_{IND}+\varepsilon_{i,t}$$ (3)

Among them, following the practice of Yang, Yin [63], the degree of shareholder power balance (*SPB*) represents the degree of balance between non-state-owned shareholders’ and state-owned controlling shareholders’ shareholder power among the top ten shareholders, which is taking the shareholding ratio of non-state-owned shareholders (*NONSTATE*) and the shareholding ratio of state-owned controlling shareholders (*STATE*) to measure (If *NONSTATE≤STATE*, *SPB = NONSTATE/STATE*; otherwise, *SPB = STATE /NONSTATE*). The degree of board power balance (*DPB*) represents the balance between non-state-owned shareholders’ and state-owned controlling shareholders’ board power among the top ten shareholders. It uses the proportion of directors appointed by non-state-owned shareholders (*NONSOE_D*) and the proportion of directors appointed by state-owned controlling shareholders (*STATE_D*) to measure (If *NONSOE_D≤STATE_D*, *DPB = NONSOE_D/ STATE_D*; otherwise, *DPB = STATE_D /NONSOE_D*). If the coefficient *α*_1_ in the model (2) and model (3) is significantly greater than 0, the degree of shareholder power balance and board power balance is significantly positively correlated with enterprise strategy; that is, there is a balance effect between the same power of heterogeneous shareholders.

Table 10 lists the balance effect test results. Column (1) shows that the degree of shareholder power balance (*SPB*) is significantly positively correlated with enterprise strategy (*SP*) (*α*_1_ = 1.084, *P*<0.01), and there is a balance effect between the shareholder power of heterogeneous shareholders. It declares that the participation of non-state-owned shareholders can indeed supervise and balance the state-owned controlling shareholders, reduce government intervention in strategic decision-making, and urge SOEs to make strategic decisions that are conducive to the long-term development of enterprises. Column (2) displays that the degree of board power balance (*DPB*) has a significant positive correlation with enterprise strategy (*SP*) (*α*_1_ = 0.849, *P*<0.05), and there is also a balance effect between the board power of heterogeneous shareholders. It explains that after non-state-owned shareholders obtain board seats, they can supervise directors and managers representing the interests of state-owned controlling shareholders, weaken the strategic defensive behaviors within SOEs due to political promotion and interest encroachment, and drive SOEs to conduct prospector strategies actively. Hypothesis H5 is verified.

**Table 10:** The test of the balance effect mechanism.

*Y = SP*	(1)	(2)
*SPB*	1.084***	
	(3.04)	
*DPB*		0.849**
		(2.23)
*CONSTANT*	27.830***	27.360***
	(10.33)	(10.23)
*CONTROLS*	*YES*	*YES*
*YEAR F*.*E*.	*YES*	*YES*
*IND F*.*E*.	*YES*	*YES*
*N*	7976	7976
*adj*. *R*^*2*^	0.144	0.140

Note: The t-statistics are reported in parentheses on robust standard errors clustered at the firm level.

*, ** and *** designate statistical significance at the 10%, 5%, and 1% level, respectively.

#### 4.4.2. Synergy effect

After examining the balance effect between heterogeneous shareholders with the same power, we also need to concentrate on whether the shareholder power and the board power owned by non-state-owned capital can interact and complement each other in strategy formulation. Referring to the research of Zhang et al. [40], models (4) and (5) are constructed to test whether there is a synergy effect between the board power of non-state-owned shareholders and the shareholder power of non-state-owned shareholders, which will have an impact on the SOEs’ strategic choice.


$${SP}_{i,t+1}=\alpha_{0}+\alpha_{1}{MIXO}_{i,t}+\alpha_{2}{NONSOE\_D}_{i,t}+\alpha_{3}{MIXO}_{i,t}\times {NONSOE\_D}_{i,t}+{CONTROLS}_{i,t}+\eta_{YEAR}+\eta_{IND}+\varepsilon_{i,t}$$ (4)

$${SP}_{i,t+1}=\alpha_{0}+\alpha_{1}{MIXO}_{i,t}+\alpha_{2}{NONSOE\_OD}_{i,t}+\alpha_{3}{MIXO}_{i,t}\times {NONSOE\_OD}_{i,t}+\alpha_{4}{CONTROLS}_{i,t}+\eta_{YEAR}+\eta_{IND}+\varepsilon_{i,t}$$ (5)

Among them, the interaction of the shareholding proportion of non-state-owned shareholders (*MIXO*) and the proportion of directors appointed by non-state-owned shareholders (*NONSOE_D*), the interaction of the shareholding proportion of non-state-owned shareholders (*MIXO*), and the proportion of directors over-appointed by non-state-owned shareholders (*NONSOE_OD*) represent the synergy effect between different powers of non-state-owned capital. If the coefficient *α*_3_ in the model (4) and (5) are significantly greater than 0, there is a synergy effect between shareholder power and board power of non-state-owned shareholders on SOEs’ strategic choices; otherwise, there is no synergy effect.

Table 11 lists the synergy effect test results. Column (1) is the regression result of non-state-owned shareholders appointing directors to represent the board power. It illustrates that the shareholder power (*MIXO*) and board power (*NONSOE_D*) of non-state-owned shareholders are significantly positively correlated with enterprise strategy (*α*_1_ = 1.825, *P*<0.01; *α*_2_ = 2.000, *P*<0.05), but the interaction coefficient is not significant (*α*_3_ = 15.660, *P*>0.1), indicating no synergy effect between different powers of non-state-owned shareholders. Column (2) is the regression result of non-state-owned shareholders over-appointing directors to represent the board power. It declares that the shareholder power (*MIXO*) and board power (*NONSOE_OD*) of non-state-owned shareholders are significantly positively correlated with enterprise strategy (*α*_1_ = 2.289, *P*<0.01; *α*_2_ = 0.713, *P*<0.05), and the interaction coefficient is also significantly positive (*α*_3_ = 6.724, *P*<0.1), stating that there is a synergy effect between different powers of non-state-owned shareholders. Hypothesis H6 is verified.

**Table 11:** The test of the synergy effect mechanism.

*Y = SP*	(1)	(2)
*MIXO*	1.825***	2.289***
	(3.70)	(4.90)
*NONSOE_D*	2.000**	
	(2.06)	
*MIXO×NONSOE_D*	15.660	
	(1.42)	
*NONSOE_OD*		0.713**
		(2.19)
*MIX×NONSOE_OD*		6.724*
		(1.65)
*CONSTANT*	28.260***	28.570***
	(24.62)	(24.94)
*CONTROLS*	*YES*	*YES*
*YEAR F*.*E*.	*YES*	*YES*
*IND F*.*E*.	*YES*	*YES*
*N*	7976	7976
*adj*. *R*^*2*^	0.144	0.143

Note: The t-statistics are reported in parentheses on robust standard errors clustered at the firm level.

*, ** and *** designate statistical significance at the 10%, 5%, and 1% level, respectively.

### 4.5. Heterogeneous group analysis

The impact of mixed-ownership reform on the SOEs’ strategic choice is not homogeneous. Due to the catalysis of the new round reform system, the external regulatory environment of SOEs is constantly changing, and there are differences in the embedded industrial environment and their characteristics. It determines that even if the shareholder power and board power of non-state-owned capital are at the same level, the inclination of SOEs to make strategic choices will be different. Therefore, this part will further explore the situational mechanism that affects the strategic choice of enterprises under the implementation of mixed-ownership reform from external supervision, industry competition, and administrative level.

#### 4.5.1. Heterogeneity test of external supervision

External supervision is vital for SOEs to convey their operating conditions to external investors and the public [64]. From the supervision mechanism, the balance effect of non-state-owned shareholders on the tunneling behaviors of state-owned controlling shareholders is further strengthened under the pressure of external public opinion and analysts’ forecasts, which reduces the possibility of collusion between non-state-owned shareholders and state-owned controlling shareholders to form an interest alliance, and enhances the role of non-state-owned shareholders in promoting the prospector strategy in line with their interest demands. In terms of reputation mechanism, the strategic adjustments made by SOEs in response to the external environment changes will be transmitted by analysts or media to external investors. The transformation of SOEs to prospectors reflects the information that enterprises can actively respond to market changes, helps to improve the social reputation and brand image of enterprises, and intensifies the motivation of non-state-owned shareholders to choose prospector strategy. In addition, the high risk of the prospector strategy increases the possibility of investment failure. Once the investment fails, it may damage the enterprise’s reputation. However, as external independent third-party intermediaries, analysts and media maintain independence, professionalism, and objectivity in formulating, implementing, and interpreting enterprise strategies [65]. It enables investors to gain a more accurate and comprehensive understanding of strategy-related information, prevents directors representing the interests of non-state-owned shareholders from being misunderstood by pursuing high-risk prospector strategies, and mobilizes the enthusiasm of non-state-owned shareholders and their appointed directors to participate in prospector strategic decision-making. Therefore, when the external supervision environment faced by SOEs is perfect, the positive impact of mixed-ownership reform on the strategy choice is more obvious.

This paper analyzes the role of external supervision from the analyst coverage and media coverage. For analyst coverage, we draw lessons from the practices of Chen et al. [65], take the median number of analysts tracked as the benchmark, the sample is divided into groups with high analyst coverage and low analyst coverage. For media coverage, referring to the practice of Meng et al. [66], and by taking the median number of annual online financial news of the company as the benchmark, the sample is divided into the group with high media coverage and low media coverage. Panel A and Panel B in Table 12 report the group test results of analyst coverage and media coverage, respectively. The positive correlation between the diversity of mixed shareholders, the depth of mixed equity, the control of mixed equity, and the excess control of mixed equity and enterprise strategy is stronger when the analyst coverage and media coverage are higher, and all of them pass the inter-group coefficient difference test. The above results show that the impact of mixed-ownership reform on prospector strategies depends on the external supervision environment faced by SOEs.

**Table 12:** The group inspection results of external supervision.

Panel A: analyst coverage								
	(1)	(2)	(3)	(4)	(5)	(6)	(7)	(8)
*Y = SP*	High	Low	High	Low	High	Low	High	Low
*MIXS*	3.252***	1.015*						
	(4.21)	(1.71)						
*MIXO*			3.671**	0.592				
			(2.41)	(0.55)				
*NONSOE_D*					4.606***	1.772		
					(2.67)	(1.30)		
*NONSOE_OD*							1.321**	0.480
							(2.15)	(0.99)
*CONSTANT*	27.30***	35.48***	29.07***	36.00***	27.91***	35.72***	28.43***	35.94***
	(7.72)	(8.66)	(8.07)	(8.87)	(7.81)	(8.75)	(7.86)	(8.84)
*CONTROLS*	*YES*	*YES*	*YES*	*YES*	*YES*	*YES*	*YES*	*YES*
*YEAR F*.*E*.	*YES*	*YES*	*YES*	*YES*	*YES*	*YES*	*YES*	*YES*
*IND F*.*E*.	*YES*	*YES*	*YES*	*YES*	*YES*	*YES*	*YES*	*YES*
*N*	3648	4328	3648	4328	3648	4328	3648	4328
*adj*. *R*^*2*^	0.211	0.130	0.202	0.128	0.200	0.129	0.197	0.128
*Diff*	-2.237*** (p = 0.000)		-3.079*** (p = 0.000)		-2.835*** (p = 0.000)		-0.841*** (p = 0.000)	
Panel B: media coverage								
	(1)	(2)	(3)	(4)	(5)	(6)	(7)	(8)
*Y = SP*	High	Low	High	Low	High	Low	High	Low
*MIXS*	2.958***	1.653***						
	(4.25)	(2.81)						
*MIXO*			2.951**	2.253*				
			(2.16)	(1.93)				
*NONSOE_D*					5.152***	2.602**		
					(3.07)	(2.03)		
*NONSOE_OD*							1.576***	0.226
							(2.68)	(0.47)
*CONSTANT*	24.81***	31.13***	25.94***	31.65***	25.14***	30.80***	25.35***	31.10***
	(7.14)	(8.86)	(7.36)	(9.03)	(7.20)	(8.75)	(7.22)	(8.86)
*CONTROLS*	*YES*	*YES*	*YES*	*YES*	*YES*	*YES*	*YES*	*YES*
*YEAR F*.*E*.	*YES*	*YES*	*YES*	*YES*	*YES*	*YES*	*YES*	*YES*
*IND F*.*E*.	*YES*	*YES*	*YES*	*YES*	*YES*	*YES*	*YES*	*YES*
*N*	3997	3979	3997	3979	3997	3979	3997	3979
*adj*. *R*^*2*^	0.179	0.127	0.170	0.125	0.170	0.124	0.167	0.122
*Diff*	-1.305*** (p = 0.000)		-0.699* (p = 0.070)		-2.550*** (p = 0.000)		-1.350*** (p = 0.000)	

Note: The t-statistics are reported in parentheses on robust standard errors clustered at the firm level.

*, ** and *** designate statistical significance at the 10%, 5%, and 1% level, respectively.

#### 4.5.2. Heterogeneity test of the degree of industry competition

In addition to the external regulatory environment, the degree of industry competition also affects the motivation of heterogeneous shareholders and directors to make strategic decisions in SOEs. Firstly, compared with the SOEs in monopoly industries, the SOEs in competitive industries face greater market competition pressure [67]. High-intensity competition often puts them under high pressure, and they always face the threat of competitors and bankruptcy. At this time, enterprises need to be flexible, innovative, and creative to deal with fierce market competition and ongoing changing competition rules [68]. Prospector strategy puts innovation and flexibility in the first place. Meanwhile, it is accompanied by high risk and high return, similar to pursuing resources and profits by non-state-owned capital. When it is hard to find market gaps in defender strategy, non-state-owned capital tends to choose prospector strategy to innovate enterprises and create new competitive advantages constantly. It is relatively easy for non-state-owned capital and their appointed directors to enter SOEs in competitive industries. They can actively participate in the SOEs’ strategic decision-making by obtaining board seats. The SOEs in monopoly industries get excess monopoly returns by government regulation, and the threshold for non-state-owned capital to enter is high [69]. Even if they can enter, their appointed directors have limited influence on the strategic decisions of SOEs. Therefore, concerning SOEs in monopoly industries, non-state-owned capital and their appointed directors of SOEs in competitive industries have stronger motivation and ability to reconstruct competitive advantages by choosing prospector strategies.

For the division of industry competition, this paper uses Yue et al. [70] for reference. It selects the coal mining and washing industry, oil and gas mining industry, etc., as monopoly industries, and the rest are defined as competitive industries. According to the industry of SOEs, we divide the samples into the group with low industry competition and the group with high industry competition. Table 13 reports the group test results of industry competition. The diversity of mixed shareholders, the depth of mixed equity, and the excess control of mixed equity are significantly positively correlated with the enterprise strategy of SOEs in competitive industries and have passed the inter-group coefficient difference test. The control of mixed equity is positively correlated with enterprise strategy in both competitive and monopolistic SOEs. Still, it is stronger in SOEs in competitive industries. It has also passed the inter-group coefficient difference test, illustrating that the industry environment of SOEs is a vital factor affecting the mixed-ownership reform and promoting the transformation of enterprises to prospectors.

**Table 13:** The group inspection results of industry competition.

	(1)	(2)	(3)	(4)	(5)	(6)	(7)	(8)
*Y = SP*	Monopoly	Competition	Monopoly	Competition	Monopoly	Competition	Monopoly	Competition
*MIXS*	2.198	2.361***						
	(1.54)	(4.23)						
*MIXO*			4.427	2.310**				
			(1.60)	(2.09)				
*NONSOE_D*					6.364*	3.443**		
					(1.81)	(2.58)		
*NONSOE_OD*							1.160	0.920**
							(1.12)	(2.06)
*CONSTANT*	28.42***	26.83***	30.27***	27.33***	28.41***	26.37***	28.30***	26.67***
	(5.24)	(8.13)	(5.44)	(8.23)	(5.19)	(8.00)	(5.17)	(8.08)
*CONTROLS*	*YES*	*YES*	*YES*	*YES*	*YES*	*YES*	*YES*	*YES*
*YEAR F*.*E*.	*YES*	*YES*	*YES*	*YES*	*YES*	*YES*	*YES*	*YES*
*IND F*.*E*.	*YES*	*YES*	*YES*	*YES*	*YES*	*YES*	*YES*	*YES*
*N*	1466	6510	1466	6510	1466	6510	1466	6510
*adj*. *R*^*2*^	0.173	0.150	0.174	0.142	0.173	0.143	0.168	0.141
*Diff*	0.164* (p = 0.060)		-2.116*** (p = 0.000)		-2.921*** (p = 0.000)		-0.240* (p = 0.050)	

Note: The t-statistics are reported in parentheses on robust standard errors clustered at the firm level.

*, ** and *** designate statistical significance at the 10%, 5%, and 1% level, respectively.

#### 4.5.3. Heterogeneity test of the degree of administrative level

Due to the different administrative levels of SOEs, the impact of government intervention on enterprise behavior decisions is disparate [55]. There are also distinctions in the motivation and ability of non-state-owned shareholders to participate in the strategic decisions of SOEs. In central SOEs, the decision-making process generally involves multiple interests. State-owned shareholders control the personnel appointment and removal, and the government has stronger intervention in these SOEs, and the reform is relatively tricky [71]. Even if non-state-owned shareholders have a certain proportion of shareholding and appointed directors, their status in SOEs is still inferior. However, for local SOEs, non-state-owned shareholders are less constrained and hindered in entering enterprises. Driven by their profit-seeking nature, non-state-owned shareholders and their appointed directors have sufficient motivation to supervise insiders and state-owned controlling shareholders to prevent SOEs’ managers from focusing only on policy tasks and avoiding the risks of choosing prospector strategies. Moreover, local SOEs obtain less economic and political resources than central SOEs and are not all in the leading position in the industry. At this time, non-state-owned shareholders need to develop new products and new markets actively, seek development opportunities to become leaders in the industry, and have a strong desire to carry out prospector strategies. Therefore, the mixed-ownership reform plays a stronger role in propelling the prospector strategy of such SOEs.

For the judgment of the administrative level of SOEs, referring to the practice of Fang [72], the samples are divided into central-controlled SOEs and local-controlled SOEs according to the type of ultimate controller. Table 14 reports the group test results at the administrative level. The diversity of mixed shareholders, the depth of mixed equity, the control of mixed equity, and the excess control of mixed equity all have a stronger positive correlation with enterprise strategy in local SOEs. All have passed the inter-group coefficient difference test. The above results state that the mixed-ownership reform implemented in local SOEs has a more significant impetus for enterprises to choose prospector strategies.

**Table 14:** The group inspection results of administrative level.

	(1)	(2)	(3)	(4)	(5)	(6)	(7)	(8)
	Central	Local	Central	Local	Central	Local	Central	Local
*MIXS*	1.376	3.005***						
	(1.48)	(4.87)						
*MIXO*			-0.367	4.487***				
			(-0.22)	(3.71)				
*NONSOE_D*					2.914	4.265***		
					(1.24)	(3.13)		
*NONSOE_OD*							0.919*	0.824**
							(1.67)	(2.10)
*CONSTANT*	30.38***	24.74***	29.98***	26.03***	30.02***	24.54***	30.22***	24.81***
	(7.26)	(7.12)	(7.06)	(7.42)	(7.20)	(6.96)	(16.38)	(16.67)
*CONTROLS*	*YES*	*YES*	*YES*	*YES*	*YES*	*YES*	*YES*	*YES*
*YEAR F*.*E*.	*YES*	*YES*	*YES*	*YES*	*YES*	*YES*	*YES*	*YES*
*IND F*.*E*.	*YES*	*YES*	*YES*	*YES*	*YES*	*YES*	*YES*	*YES*
*N*	2646	5330	2646	5330	2646	5330	2646	5330
*adj*. *R*^*2*^	0.168	0.166	0.165	0.160	0.167	0.154	0.166	0.151
*Diff*	1.629*** (p = 0.000)		4.854*** (p = 0.000)		1.352*** (p = 0.000)		-0.094 (p = 0.250)	

Note: The t-statistics are reported in parentheses on robust standard errors clustered at the firm level.

*, ** and *** designate statistical significance at the 10%, 5%, and 1% level, respectively.

## 5. Conclusions

This paper studies the impact of mixed-ownership reform on the SOEs’ strategic choice from the perspective of dual control rights of shareholder power and board power. The main research conclusions are as follows: (1) The diversity of mixed shareholders, the depth of mixed equity, the control of mixed equity, and the excess control of mixed equity are significantly positively correlated with the enterprise strategy, manifesting that the higher the degree of SOEs’ mixed-ownership reform, the more likely they are to choose the prospector strategy. (2) The mechanism test finds that mixed-ownership reform can affect enterprises’ strategic choices through the balance effect between non-state shareholders and state-controlling shareholders with dual control rights. And when the proportion of over-appointed directors by non-state-owned shareholders represents the board power, the mixed-ownership reform can also play a governance role through the synergy effect between different powers of non-state-owned shareholders. (3) Among the SOEs under strict external supervision, competitive industries, and local areas, the mixed-ownership reform can better promote the strategy transformation of SOEs to prospectors.

This paper has the following policy implications: (1) The decision-makers of SOEs should unswervingly implement mixed-ownership reform, ensure that external strategic investors have both shareholder power and board power in SOEs, and promote in-depth cooperation between state-owned shareholders and non-state-owned shareholders. Non-state-owned shareholders with market-oriented governance logic can give full play to their market initiative through mixed-ownership reform, improve the internal governance system, ameliorate the strategic decision preferences and processes of the board, formulate strategic goals that match the external environment and form strategic-oriented operating model to improve the SOEs’ operating vitality. (2) The mixed-ownership reform of SOEs should "adapt to enterprise conditions" and promote the hierarchical and classified reform while perfecting the external supervision system. The State-owned Assets Supervision and Administration Commission of the State Council of governments can formulate different reform plans according to the type of enterprise. Competitive SOEs and local SOEs are subject to less government intervention. They mainly impulse the policy of SOEs to cultivate market players to speed up the reform process. For SOEs in monopoly industries, it is necessary to continuously open government regulations such as industry access and develop the competitive business of enterprises. Meanwhile, it is also vital to play the exemplary leading role of central SOEs, optimize the internal management system and operation mechanism, propel enterprises to carry out prospector strategies, and achieve the high-quality development of enterprises and the economy.

Although we have tried our best to make theoretical contributions and implications, there are still some limitations to be improved. In terms of indicator measurement, we mainly refer to the strategic classification method of Miles, Snow [20], and classify the enterprises’ strategic types accordingly. In the future research, we can consider broadening the strategic classification framework, dividing strategies from the business characteristics, geographical location, etc., investigating the impact of mixed-ownership reform on different types of SOEs’ strategic positioning, and verifying the overall strategic adjustment direction after the SOEs’ mixed-ownership reform. In terms of research samples, this paper selects state-owned listed companies whose data are publicly available for research. However, in China’s state-owned non-listed companies, non-state-owned shareholders may also hold certain shares and assign key personnel to the enterprises, exerting a positive impact on the strategic behaviors of SOEs. As it is difficult to obtain the data of non-listed companies, the data of state-owned non-listed companies will be collected manually for subsequent verification in the future.

## Supporting information

S1 Data (XLSX)
